# Associations between olfactory reference disorder and social phobia – results of an internet-based study

**DOI:** 10.3389/fpsyg.2024.1248496

**Published:** 2024-03-07

**Authors:** Julia Reuter, Anja Grocholewski, Regina Steil

**Affiliations:** ^1^Department of Clinical Psychology and Psychotherapy, Institute of Psychology, Goethe University Frankfurt, Frankfurt am Main, Germany; ^2^Department of Clinical Psychology, Psychotherapy, and Diagnostics, Technische Universität Braunschweig, Braunschweig, Germany

**Keywords:** olfactory reference disorder, ICD-11, social phobia, social anxiety disorder, avoidance, insight, shame

## Abstract

Despite the similar clinical features of Olfactory Reference Disorder (ORD) and Social Phobia (SP), or studies showing elevated comorbidity of the two disorders, and the conceptualization of ORD as a form of SP in the East Asian culture, to our knowledge, the relationship between ORD and SP has not been investigated. This study examined the association of ORD according to the 11th revision of the International Classification of Diseases (ICD-11) and SP in 225 German university / college students who completed self-ratings with regard to socio-demographic data and symptoms of SP and ORD within an anonymous internet-based survey. Symptoms of SP were assessed with the Social Phobia Inventory (SPIN). Symptoms of ORD according to the ICD-11 were assessed with the Olfactory Reference Disorder Questionnaire (ORDQ), developed for this study. In our sample, 86.6% of the participants who met the self-rated features for ORD also met the self-rated criteria for current SP. ORD severity scores were significantly related to SP. Participants with and without self-reported ORD differed significantly in their SP total scores. SP severity was also significantly correlated with poorer insight of ORD-related beliefs, greater ORD-related avoidance of intimate relationships and higher levels of shame and fear of rejection due to body odor. These preliminary findings indicate that ORD could be closely related to SP and highlight the need for future research on the relationship of ORD and SP in order to gain a better understanding of the development, maintenance, treatment and classification of ORD.

## 1 Introduction

Olfactory reference disorder (ORD) is a distressing and impairing condition characterized by a persistent preoccupation with the belief that one is emitting a foul or offensive body odor or breath that is usually not perceptible to others (World Health Organization, 2019). Research studies examining the prevalence of ORD in university students suggest that the disorder is relatively common - estimates of its prevalence vary between 2.1 and 5.5% (Kasahara and Kenji, 1971; Zhou et al., 2018; Reuter et al., 2023). However, a systematic examination of its prevalence in the general population is lacking. Indeed, empirical research on ORD is scarce and most findings on this disorder are limited to case reports or case series with only a few subjects. However, the suffering of those affected (e.g., Feusner et al., 2010; Prazeres et al., 2010; Greenberg et al., 2016) underscores the importance of investigations into the disorder.

Recently, ORD has been included as a new diagnosis in the category of obsessive-compulsive and related disorders in the 11th revision of the International Classification of Diseases (ICD-11) (World Health Organization, 2019). However, although ORD has now been assigned as being part of the obsessive-compulsive disorder (OCD), its classification is still a matter of debate. There are similarities to OCD insomuch as individuals with ORD perform repetitive behaviors and are preoccupied with permanent thoughts about their odor. Moreover, OCD is a common comorbid disorder of ORD which further supports their overlap. Specifically, comorbidity rates of 25–50% have been reported in clinical samples consisting of 20 (Phillips and Menard, 2011) and 14 (Prazeres et al., 2010) patients with diagnosed ORD. Based on case reports, similarities of the two conditions are also evident in their pharmacological and psychotherapeutic treatment responses (e.g., Stein et al., 1998; Phillips and Castle, 2007; Martin-Pichora and Antony, 2011). However, individuals with OCD are usually aware of the exaggeration and senselessness of their actions or fears (Eisen et al., 2004; Schulte et al., 2020), whereas this does not seem to be the case with the majority of those affected by ORD since insight is poorer in ORD (previous studies report percentages of 57–90%; Begum and McKenna, 2011; Phillips and Menard, 2011; Zhou et al., 2018) than in OCD (percentages of 2% are reported; Eisen et al., 2004; Phillips et al., 2007). In addition, the prevalence of ideas of reference is higher in individuals with ORD as compared to OCD (Kozak and Foa, 1994; Feusner et al., 2010; Phillips and Menard, 2011; Greenberg et al., 2016) and, though data are limited, research suggests the higher co-occurrence of social phobia (SP) and major depressive disorder in ORD (Prazeres et al., 2010; Phillips and Menard, 2011) compared to OCD (Denys et al., 2004; Torresan et al., 2013; Lochner et al., 2014).

Apart from OCD, clinical observations and available data show an overlap of ORD with features of SP. For instance, fears of negative evaluation and rejection as well as social avoidance also seem to be present among those affected with ORD (Phillips and Menard, 2011; Veale and Matsunaga, 2014; Greenberg et al., 2016). Greenberg et al. (2016) found that most (80%) of their 253 subjects with ORD reported that they feared that their odor could offend others. Moreover, in their sample of 38 persons with diagnosed ORD who went through qualitative interviews, Schmidt et al. (2022) found that it seems to be predominantly social situations that trigger ORD symptoms. Thereby, the literature suggests that individuals affected by ORD fear being rejected or humiliated by others because of their smell, that social situations are endured with the use of safety behaviors similar to SP, and that similar feelings to those in SP, namely shame and anxiety, arise in social situations if these cannot be avoided (Pryse-Phillips, 1971; Liebowitz et al., 1985; Feusner et al., 2010; Stein et al., 2016; Swee et al., 2021; Schmidt et al., 2022). Another similar feature in the psychopathology of ORD with SP appears to be the awareness of self-referent information. ORD is characterized by an excessive self-consciousness about the perceived odor (World Health Organization, 2019). Such a consciousness of the self is also found in SP where those affected divert their attention to excessive self-monitoring in social situations (Clark and Wells, 1995). This self-focused attention in SP has been proposed to be an important maintaining factor of the disorder (Clark and Wells, 1995). In addition, SP has been found to be a common comorbid disorder in patients with ORD, and this supports a possible relationship between the two disorders. In their patient sample, Phillips and Menard (2011) found that 65% of their 20 patients had a lifetime diagnosis of SP while 60% had a current SP. Due to such a high occurrence of SP, the authors suggested that patients with ORD should also be carefully assessed for SP (Phillips and Menard, 2011). Similarly, in their assessment of 106 OCD patients and 65 SP patients, Lochner and Stein (2014) found a higher prevalence of ORD in SP than in OCD patients.

Indeed, the Diagnostic and Statistical Manual of Mental Disorders (DSM-5) even refers to the proximity of SP and ORD, although ORD has not been defined as a separate diagnosis within it. Specifically, ORD is described in the DSM-5 under “*Jikoshu-kyofu*,” a condition in the Japanese diagnostic system defined as the fear of having an offensive body odor (American Psychiatric Association, 2013). “*Jikoshu-kyofu*” is a variant form of the culturally bound syndrome “*taijin kyofusho*,” a disorder of interpersonal fear and a type of SP in Eastern cultures (Kleinknecht et al., 1997; American Psychiatric Association, 2013).

In light of the previous findings and the conceptualization of ORD in the East Asian culture which suggest a link between ORD and SP, it is surprising, and apparently due to the aforementioned blatant lack of studies, that this relationship has not been empirically investigated. There are only a few studies that have assessed just single symptoms of SP among individuals with ORD, while there is one report from Phillips and Menard (2011) that has assessed solely the comorbidity of SP in their 20 patients with ORD. In addition, it is worth mentioning that due to a lack of standardized diagnostic criteria before the ICD-11, ORD was assessed using different diagnostic criteria in earlier studies.

Therefore, the present study examined the association of ICD-11 ORD and SP in university students, a convenient, nonclinical sample. To our knowledge, this is the first investigation to examine correlates of SP in participants reporting ORD according to the ICD-11. Based on the literature and our clinical experience, we hypothesized that ORD severity would be positively associated with SP and that higher levels of SP would be related to poorer insight (delusionality) of ORD-related beliefs, greater ORD-related avoidance of intimate relationships and higher levels of shame and fear of rejection due to body odor.

## 2 Methods

### 2.1 Sampling and procedure

The data used in this study are part of a larger research assessment focusing on epidemiological aspects and correlates of ORD (see also Reuter et al., 2023) which was completed through an anonymous internet-based survey using the survey tool provided by SoSci Survey (Leiner, 2019). We decided to conduct an anonymous online survey since ORD is accompanied by shame and avoidance of social contacts (e.g., Phillips and Menard, 2011; Schmidt et al., 2022). An anonymous online survey therefore seemed favorable to reach as many severely affected people as possible who might not reveal their worries in personal contact out of shame. Inclusion criteria comprised being aged 18 years or older and being a student at a university or college. Participants were recruited by an advertisement redirecting readers to the survey. The advertisement was placed on the websites, social media platforms and campus e-mail newsletters of the Goethe-University (GU) Frankfurt, the Technische Universität (TU) Braunschweig and adjoining universities and colleges between December 2021 and May 2022 (until a sufficient sample size was reached). Potential participants were invited to an anonymous online survey which would take between about 5 and 30 min to complete, depending on the response behavior (the assessment was shortened for participants who reported no symptoms of ORD [score = 0, “*not at all*”] as assessed via the first five items of the *Olfactory Reference Disorder Questionnaire* [ORDQ, see 2.2.2] which was presented at the beginning of the assessment. If no ORD symptoms were reported, the remaining questionnaires, following the ORDQ, were dropped). It was stated that the results of the study might help to improve the care of people suffering from an extreme preoccupation with the belief of emitting a foul or offensive body odor. In order to increase participation, we provided an incentive by raffling two €500 online shopping gift cards upon the completion of the assessment. Participants were informed about all aspects of the study in order to make an informed decision about participation, including information about the aim, procedure, scope, possible risks of the study, data protection and that they were free to withdraw from the study at any time without giving reasons. Informed consent was presented on a cover page before starting the survey; only if the participants consented to participate were they directed to the survey. The study was approved by the ethics committee of the Medical Faculty of Goethe-University Frankfurt and by the ethics committee of Technische Universität Braunschweig.

### 2.2 Measures

The measures were administered in German.

#### 2.2.1 Socio-demographic data

We assessed age, sex, relationship status, level of education and bodily diseases that may cause malodor (hyperhidrosis, trimethylaminuria, gastroesophageal reflux disease, achalasia, irritable bowel syndrome, periodontitis, or extreme tooth decay, as well as tumors in the mouth, throat, nose, or gullet).

#### 2.2.2 Olfactory reference disorder questionnaire (ORDQ)

The ORDQ is a 16-item self-report measure assessing the ICD-11’s essential features of ORD (persistent preoccupation, self-consciousness, repetitive and/or avoidance behaviors, distress and/or impairment) over the past three months; we had to develop this as such since there was no existing questionnaire assessing ORD according to the ICD-11. Furthermore, the current version of ICD-11 does not specify a minimum duration to diagnose ORD. Therefore, since we assumed that one cannot consider “persistent” symptoms as being less than three months’ duration and to ensure as high a reliability as possible, we decided, after also consulting three international experts on ORD, on a three-month history of ORD symptoms. Each item is rated on a 5-point Likert scale (“0” = not at all, “1” = a little, “2” = moderate, “3” = severe, “4” = extremely).

The first four items of the questionnaire can be used as screening items since they assess the essential features of ICD-11 ORD as described above (1: *“I am persistently preoccupied about the fact that I emit, or could emit, a foul or offensive body odor or breath,” 2: “I am excessively self-conscious about my body odor or breath,” 3: “Due to the concern about my body odor or breath, I carry out repeated, excessive behaviors (e.g., I repeatedly check my body odor or repeatedly ask other people about my smell, repeatedly apply perfume or deodorant, I shower several times a day) or avoid (social) situations or activities,” and 4: “I suffer from the concern about my body odor or breath or am impaired in my life”*).

Insight into ORD beliefs is assessed by the fifth item of the ORDQ *(“I am absolutely convinced that one or more parts of my body (*e.g.*, mouth, hands, feet, chest, genital area,..) exude an unpleasant smell, although other people have assured me that this is not the case”)*. The insight of the participants was measured in this study with this item.

The remaining 11 subitems ask in-depth questions. The total score of ORD symptom severity is obtained by summing up the scores of items 1–16, resulting in a score range from 0 to 64. In the present study, participants with a score greater than or equal to 2 on all four screening items (1–4) were considered to have a probable diagnosis of ORD. Internal consistency (Cronbach’s alpha) of the total scale for the present sample was 0.92 and for the first four screening items was 0.85.

#### 2.2.3 ORD-related avoidance of intimacy, shame, and fear of rejection

Shame and fear of rejection as well as the avoidance of intimate relationships due to one’s body odor were assessed by two additional items to the ORDQ. Both features are often described in the literature as symptoms of ORD (e.g., Feusner et al., 2010; Greenberg et al., 2016), but are not included in the ICD-11 description of ORD.

#### 2.2.4 Social phobia inventory (SPIN)

Social phobia was assessed by the German version of the SPIN (Connor et al., 2000; Stangier and Steffens, 2002). This consists of 17 items evaluating fear, avoidance and physiological discomfort in the previous week. Items are rated on a 5-point Likert scale (“0” = not at all, to “4” = extremely). The total score ranges from 0 to 68. A cut-off score ≥ 25 in the German version has been proposed to suggest a social anxiety disorder (Sosic et al., 2008). The SPIN has shown good psychometric properties (Sosic et al., 2008). Internal consistency (Cronbach’s alpha) in the present study was 0.94 (Connor et al., 2000).

#### 2.2.5 Data analysis

Analyses were run using IBM SPSS version 28 (SPSS Inc., Chicago, United States). Pearson product–moment correlation coefficients examined the relationship between ORS and SP total symptom severity. Spearman’s rank correlation examined the relationship between the remaining selected variables. Between-group differences were conducted using non-parametric Mann–Whitney U-tests. Results were evaluated using 95% confidence intervals (CIs). The significance threshold for all analyses was set at *p* < 0.05. Missing values were avoided in advance by using the forced choice format.

## 3 Results

### 3.1 Sample description

A total of 440 students accessed the survey; of these, 404 participants gave informed consent. A total of 275 participants completed the survey of whom 15 participants screened positive for self-reported ORD (five participants were excluded since they reported a medical condition that might account for malodor; for a detailed description on the prevalence estimation see Reuter et al., 2023). Since 50 participants skipped all of the questionnaires due to the complete lack of ORD symptoms being recorded in the ORDQ at the beginning of the assessment (see 2.1), the final sample consisted of 225 participants who completed the questionnaires. The majority (*n* = 177 [78.7%]) of the participants were female. The mean age was 24.76 years ± 5.28 (age range = 18–65 years). Regarding their relationship status, the majority (*n* = 88 [39.1%]) were single. Table 1 provides a detailed description of the participants’ socio-demographic information.

**Table 1:** Socio-demographic characteristics of the total sample (*n* = 225) and the ORD group (*n* = 15).

	Total sample *n* (%)	ORD group *n* (%)
*Gender*		
Female	177 (78.7)	12 (80.0)
Male	45 (20.0)	2 (13.3)
Diverse	3 (1.3)	1 (6.7)
*Relationship status*		
Single	88 (39.1)	8 (53.3)
Partnership, living together	50 (22.2)	3 (20.0)
Partnership, living apart	72 (32.0)	2 (13.3)
Married, living together	11 (4.0)	2 (13.3)
Married, living apart	3 (1.3)	0
Divorced	1 (0.4)	0
Age (years)	*M* (SD) 24.76 (5.28)	*M* (SD) 24.2 (5.36)

M, Mean; SD, Standard deviation.

### 3.2 Associations between ORD with variables of interest

The means and standard deviations for the SP and ORD variables are presented in Table 2. We examined the associations of ORD with SP, delusionality, avoidance of intimate relationships and the feelings of shame and fear of rejection. As predicted, the ORD total symptom severity was significantly positively related to SP total symptom severity (*r* = 0.53, *p* < 0.001). Of the 15 participants who met the self-rated features for ORD, nearly all had clinically significant social phobia scores: 86.6% (*n* = 13) had a SPIN score ≥ 25, indicating a current SP. In addition, the SP total scores in the SPIN of the participants with self-reported ORD (*M* = 32.46, *SD* = 11.84, 95% CI: 25.91–39.02) and without self-reported ORD (*M* = 18.71, *SD* = 14.17, 95% CI:16.79–20.64) differed significantly according to the Mann–Whitney *U*-test (*U* = 715.00, *Z* = −3.53, *p* < 0.001).

**Table 2:** Range, means, and standard deviations of the SP and ORD total scores.

		ORD group				Non-ORD group		
	*n*	Range	*M* (95% CI)	*SD*	*n*	Range	*M* (95% CI)	*SD*
SPIN	15	10–57	32.46 (25.9–39.02)	11.83	210	0–58	18.71 (16.79–20.64)	14.16
ORDQ	15	13–45	29.60 (24.16–35.03)	9.81	260	0–55	8.49 (7.41–9.57)	8.84

The sample sizes differ because 50 participants who did not report ORD symptoms in the ORDQ skipped all the following questionnaires, including the SPIN. M, Mean; SD, Standard Deviation; CI, Confidence Interval; SPIN, Social Phobia Inventory; ORDQ, Olfactory Reference Disorder Questionnaire.

As expected, SP severity was significantly positively correlated with delusional conviction (*r* = 0.40, *p* < 0.001), avoidance of intimate relationships due to malodor concerns (*r* = 0.31, *p* < 0.001), and the feelings of shame and fear of rejection due to body odor (*r* = 0.32, *p* < 0.001).

## 4 Discussion

The results suggest high rates of SP in individuals with ORD. In our sample, 86.6% of the participants who met the self-rated features for ORD also met the self-rated criteria for a current SP. This finding is consistent with previous research reporting elevated rates of SP in individuals suffering from ORD (Phillips and Menard, 2011) and shows that ORD and SP co-occur. The high rate of co-occurrence (as well as the clear phenomenological overlap) may indicate that both conditions might share underlying factors. However, future research is needed to investigate this hypothesis. At the moment, too little is known about the epidemiology as well as the factors of the origin and maintenance of ORD to make predictions. Although currently classified as an obsessive-compulsive disorder, the comorbidity rates of OCD in ORD seem to be lower: co-occurrence rates of between 25% (Phillips and Menard, 2011) and 50% (Prazeres et al., 2010) have been reported. However, given the few available studies on ORD, future research investigating the comorbidity rates of SP and OCD in ORD, in particular using clinical interviews, is needed. In addition, the findings of a higher comorbidity of SP compared to OCD in ORD leaves the question unanswered of whether ORD is more strongly associated with SP compared to OCD or whether this is due to the generally higher prevalence of SP compared to OCD (e.g., Bebbington, 1998; Stangier, 2016).

The ORD severity scores were significantly related to SP as measured by the SPIN. The association with SP determined in this study indicates that social anxiety may be related to olfactory concerns which, in turn may cause or aggravate concerns about social acceptance. In addition, the SPIN mean score of 32.46 (*SD* = 11.84) for the participants who met the self-reported features for ORD was approximately three standard deviations above the mean of 14.56 (*SD* = 9.36) for the nonclinical control group reported for the German version of the scale (Sosic et al., 2008); this indicates a higher magnitude of fear, avoidance and physiological symptoms of SP in ORD relative to nonclinical samples. Our finding of a significant difference in the SPIN total scores between participants with and without probable ORD supports this assumption. However, at the same time it is clear that our results require confirmation from further research. Due to the cross-sectional design, we are not able to determine causality. In addition, we did not investigate whether other conditions could have influenced the relationship between ORD and SP. In future studies, possible confounding variables should be assessed.

As anticipated, SP severity was significantly associated with greater ORD-related avoidance of intimate relationships and higher levels of shame and fear of rejection due to body odor. This finding is consistent with previous studies showing an overlap of the central features between SP and ORD (e.g., Phillips and Menard, 2011) and underscores the social aspect of olfactory concerns. Indeed, in light of our results and previous findings, there is much indication that both conditions share, as a central aspect, an excessive concern about the negative evaluation of others. In any event, future research investigating cognitive factors underlying ORD as well as shared mechanisms underlying ORD and SP are warranted. In this regard, a worthwhile research question could be what role do safety behaviors and self-focus play in the maintenance of ORD. As mentioned above, both are features of the disorder. In their prominent cognitive model of social phobia, Clark and Wells (1995) proposed that safety behaviors and self-focused attention are important maintaining factors in SP. The cognitive therapy of SP (Clark and Wells, 1995; Wells, 1997) based on their model focuses on a change in these two factors (e.g., shifting the attentional focus, dismissing safety behaviors) and has proven to be effective (e.g., Clark et al., 2003). If self-focused attention and safety behaviors are also key maintaining factors in ORD, the cognitive therapy of SP could possibly also be effective in the treatment of ORD.

Likewise, as hypothesized, we found that poor insight in ORD was significantly related to the SP severity scores. Poor insight is a prominent feature of ORD since the individuals affected are often convinced that their perceived body odor is real and has a physical rather than a psychological reason (Begum and McKenna, 2011; Phillips and Menard, 2011; Zhou et al., 2018). However, studies show that there is a range of different insight levels (e.g., Phillips and Menard, 2011; Greenberg et al., 2016). Moreover, in their review of case reports on ORD, Begum and McKenna (2011) report that the level of insight can also fluctuate within patients. This coincides with our clinical impression that the convictions of patients with ORD often fluctuate between obsessional thoughts, overvalued ideas or referential thinking, with some beliefs reaching a delusional quality. The relationship between poor insight and SP in our study could be explained by the overlap between delusional conviction and overvalued ideas or ideas of reference (Watzke and Schwenke, 2014) which are a common feature of SP (e.g., Konermann and Zaudig, 2003). However, further research is required to elucidate the relationship between poor insight in ORD and SP.

Our findings, demonstrating a significant association between ORD severity and SP, as well as a high comorbidity between ORD and SP, are consistent with previous studies linking ORD to features of social phobia (e.g., Phillips and Menard, 2011; Veale and Matsunaga, 2014; Schmidt et al., 2022) and indicate that the Eastern conceptualization of ORD as a type of SP deserves attention in Western research. Doubtless to say, our results are preliminary. Further research using large samples representative for the general population and especially with the use of clinical interviews to assess diagnoses is needed to investigate the relationship between ORD and SP, including their common features and shared etiologic factors. With our results, no statement can be made about whether ORD could be a subtype of SP or not. To determine this, large epidemiological studies would be necessary in which the psychopathology, comorbidity, genetic underpinnings and familial aggregation of ORD and SP are investigated. In any case, our findings suggest that this further research into the association of the two conditions could be valuable to clarify the classification of ORD.

The strength of our study is that, to our knowledge, for the first time the relationship between ORD and SP has been directly examined. Moreover, we report data on ICD-11 ORD while previous studies captured ORD with different criteria due to the absence of standardized diagnostic categorization. However, there are also significant limitations in our study. Firstly, even though one has better access to severely affected individuals by using an anonymous online survey, since ORD is accompanied by much shame (Pryse-Phillips, 1971; Stein et al., 1998), our data, therefore, relies purely on these self-ratings. Without an in-person clinical interview, no information about the diagnostic status with regard to ORD or SP can be obtained (e.g., regarding ORD, we could not determine whether there was an actual body odor or whether comorbid psychopathology may have influenced or explained the ORD symptoms). Secondly, since our sample consisted mostly of women, the results may not be generalizable to men affected by ORD. Furthermore, the generalizability of our results is limited by the fact that our sample consisted of university students. Therefore, our results are not representative for the general population. In addition, there could have been selection effects in the recruitment of the participants. Finally, although the internal consistency of the ORDQ was good (for the four screening items) to excellent (total scale), the instrument still needs to be evaluated. With regard to the validity of the ORDQ, the correlation with the SPIN suggests that discriminant validity could be given. However, against the background of the limitations of the online survey and the only small number of cases of the participants with self-reported ORD, no valid statements can be made about the psychometric properties of the instrument. Currently, there are no evaluated instruments to assess ORD and, to our knowledge, there are no other available instruments assessing ORD according to the ICD-11. Future research on ORD would benefit from the development and evaluation of instruments to assess ORD.

## 5 Conclusion

To our knowledge, this is the first study that has directly examined the relationship between ORD and SP. In our sample of university students, we found a significant positive association between self-reported SP and self-reported ORD and a high comorbidity (86.6%) of self-reported SP in individuals reporting ICD-11 ORD. Higher levels of SP were related to a poorer insight of ORD-related beliefs, greater ORD-related avoidance of intimate relationships and higher levels of shame and fear of rejection due to body odor.

In Western research, ORD has so far been mainly compared to OCD and ultimately conceptualized as an OCD spectrum disorder in the ICD-11. However, the findings of this study indicate that ORD could also be closely related to SP. If this is confirmed by future studies, treatment concepts that have proven to be successful in the treatment of SP could also be effective in the treatment of ORD.

Although preliminary, our results have implications for clinical practice, and suggest that additional research on the relationship of ORD and SP, including their possible interplay or shared etiologic factors, could be of value in order to gain a better understanding of the development, maintenance, classification and treatment of ORD.

## Data availability statement

The raw data supporting the conclusions of this article will be made available by the authors, without undue reservation.

## Ethics statement

The studies involving humans were approved by Ethics Committee of the Medical Faculty of Goethe-University Frankfurt, Germany and the Ethics Committee of Technische Universität Braunschweig, Germany. The studies were conducted in accordance with the local legislation and institutional requirements. The participants provided their online informed consent to participate in this study.

## Author contributions

JR, RS, and AG contributed to the study concept and design. JR originated the idea for this manuscript, conducted the study, collected the data and performed the statistical analysis and interpretation of data, coordinated and wrote the first draft of the manuscript. RS and AG critically revised the draft of the manuscript. All authors contributed to the article and approved the submitted version.
